# Lennox–Gastaut syndrome unveiled: Advancing diagnosis, therapies, and advocacy‐insights from the Genoa International Workshop

**DOI:** 10.1111/epi.18696

**Published:** 2025-10-29

**Authors:** Antonella Riva, Gianluca D'Onofrio, Elisabetta Amadori, Alexis Arzimanoglou, Stéphane Auvin, Irene Bagnasco, Paola Barabino, Valentina Biagioli, Isabella Brambilla, Giuliana Cangemi, Antonietta Coppola, Antonella De Lillo, Carlo Di Bonaventura, Giancarlo Di Gennaro, Edoardo Ferlazzo, Antonio Gil‐Nagel, Giuseppe Gobbi, Simona Lattanzi, Gerhard Kluger, Günter Krämer, Maria Margherita Mancardi, Carlo Minetti, Lino Nobili, Elisa Paravati, Milka Pringsheim, Erika Rebessi, Antonino Romeo, Angelo Russo, Emilio Russo, Katia Santoro, Susanne Schubert‐Bast, Laura Siri, Jo Sourbron, Maria Stella Vari, Alberto Verrotti, Flavio Villani, Maurizio Viri, Celina von Stülpnagel, Nelia Zamponi, Federico Zara, Pasquale Striano

**Affiliations:** ^1^ Department of Neurosciences Rehabilitation, Ophthalmology, Genetics, Maternal and Child Health (DINOGMI) University of Genoa Genoa Italy; ^2^ IRCCS Istituto “Giannina Gaslini”, Full Member of ERN EpiCARE Genoa Italy; ^3^ Epilepsy Unit, Child Neurology Department, Hospital San Juan de Déu, Coordinator of the ERN EpiCARE Universitat de Barcelona Barcelona Spain; ^4^ INSERM NeuroDiderot, Université Paris Cité Paris France; ^5^ Pediatric Neurology Department CRMR Epilepsies Rares, ERN EpiCare, APHP, Robert Debré University Hospital Paris France; ^6^ Institut Universitaire de France (IUF) Paris France; ^7^ Division of Child Neuropsychiatry Martini Hospital, Child Neuropsychiatry ToSud e Asl città di Torino Turin Italy; ^8^ UOC‐Unità Operativa Complessa Farmacia, IRCCS Istituto “Giannina Gaslini” Genoa Italy; ^9^ Dravet Italia Onlus Verona Italy; ^10^ Epag ERN EpiCare Verona Italy; ^11^ Research Center for Pediatric Epilepsies (CREP), Department of Surgery, Dentistry, Paediatrics and Gynecology University of Verona Verona Italy; ^12^ Alleanza Epilessie Rare e Complesse Italy; ^13^ Biochemistry, Pharmacology and Newborn Screening Unit, Central Laboratory of Analysis IRCCS Istituto Giannina, Gaslini Genoa Italy; ^14^ Epilepsy Center University Hospital Federico II Naples Italy; ^15^ Department of Human Neurosciences Policlinico Umberto I, Sapienza University of Rome Rome Italy; ^16^ IRCCS NEUROMED, Pozzilli Isernia Italy; ^17^ Department of Medical and Surgical Sciences Magna Græcia University of Catanzaro Catanzaro Italy; ^18^ Regional Epilepsy Centre, Great Metropolitan “Bianchi‐Melacrino‐Morelli Hospital” Reggio Calabria Italy; ^19^ Department of Neurology Hospital Ruber Internacional Madrid Spain; ^20^ Fundación Iniciativa Para Las Neurociencias (Fundación INCE) Madrid Spain; ^21^ Associazione Famiglie LGS Italia Correggio Italy; ^22^ Or.S.A Treviso Italy; ^23^ Associazione Sclerosi Tuberosa Rome Italy; ^24^ Neurological Clinic, Department of Experimental and Clinical Medicine Marche Polytechnic University Ancona Italy; ^25^ Department of Pediatrics, Institute of Rehabilitation, Transition and Palliation of Neurologically Ill Children Paracelsus Medical University, Member of the ERN Epicare Salzburg Austria; ^26^ Center for Pediatric Neurology, Neurorehabilitation and Epileptology, Schoen‐Clinic Vogtareuth Vogtareuth Germany; ^27^ Neurocenter Sternen in Zurich Zurich Switzerland; ^28^ Child Neuropsychiatry Unit Full Member of the ERN EpiCARE, IRCCS Istituto Giannina Gaslini Genoa Italy; ^29^ Centro di PsicoMedicina Sistemica, Valeggio Sul Mincio Verona Italy; ^30^ Schön Klinik Vogtareuth Vogtareuth Germany; ^31^ PMU Salzburg Salzburg Austria; ^32^ TUM Klinikum, German Heart Centre Munich Germany; ^33^ Pediatric Institute of Southern Switzerland, Ente Ospedaliero Cantonale Bellinzona Switzerland; ^34^ Fatebenefratelli Hospital, ASST Fatebenefratelli Sacco Milan Italy; ^35^ U.O.C. Neuropsichiatria Dell'età Pediatrica, Member of the ERN EpiCare, IRCCS Istituto Delle Scienze Neurologiche di Bologna Bologna Italy; ^36^ Department of Science of Health Magna Græcia University of Catanzaro Catanzaro Italy; ^37^ Center of Neurology and Neurosurgery, Epilepsy Center Frankfurt Rhine‐Main Goethe‐University Frankfurt Frankfurt am Main Germany; ^38^ LOEWE Center for Personalized and Translational Epilepsy Research (CePTER) Goethe‐University Frankfurt Frankfurt am Main Germany; ^39^ Department of Pediatric Epileptology, University Children's Hospital Goethe‐University Frankfurt Frankfurt am Main Germany; ^40^ Section Pediatric Neurology, Department of Development and Regeneration University Hospital KU Leuven Leuven Belgium; ^41^ Center for Medical Genetics Ghent University Hospital Ghent Belgium; ^42^ Paediatric Neurology and Muscular Disease Unit IRCCS Istituto “Giannina Gaslini”, Full Member of ERN EpiCARE Genoa Italy; ^43^ Department of Paediatrics University of Perugia Perugia Italy; ^44^ Clinical Neurophysiology Unit and Epilepsy Center IRCCS Ospedale Policlinico San Martino Genoa Italy; ^45^ Department of Child Neurology and Psychiatry AOU Maggiore Della Carità Novara Novara Italy; ^46^ Division of Pediatric Neurology, Developmental Medicine and Social Pediatrics, Department of Pediatics Ludwig Maximilians University Munich, Dr von Hauner Children's Hospital, iSPZ Hauner Munich Germany; ^47^ Child Psychiatry and Neurology Unit G. Salesi Hospital Ancona Italy

**Keywords:** epilepsy, Lennox–Gastaut, multidisciplinary care, patient advocacy

## Abstract

Lennox–Gastaut syndrome (LGS) is one of the most severe, yet one of the most discussed, childhood‐onset developmental and epileptic encephalopathies (DEEs). Dissent among epileptologists on the definition and minimum set of electroclinical features derives from the high etiological heterogeneity within the syndrome, which could make its prevalence overestimated. However, in recent years, our diagnostic strategies, including both high‐resolution magnetic resonance imaging and next‐generation sequencing techniques, have enabled us to disentangle many cases previously classified as “idiopathic.” In addition, some electroencephalographic and circulating biomarkers have been identified that could predict disease progression and treatment response if confirmed in larger patient populations. As our diagnostic capacity increases, so do our treatment strategies. Although progress has been made, the implementation of better clinical trial designs, individualized treatments, and therapies that address the genetic roots of the disease remains necessary in clinical practice. A lot is being done in this direction, thanks to the involvement of families and the creation of international networks, such as the ERNs, which are rapidly promoting collaboration among highly specialized centers and the establishment of disease registries to shed light on the natural history of LGS. Yet, many unmet needs still afflict patients and their families, including uncertainties arising from the transition process and a lack of administrative support and comprehensive care as patients transition into adulthood. This article summarizes these key challenges in diagnosing, treating, and caring for patients with LGS, as well as the roadmap to enhanced future care discussed during the international LGS meeting held in Genoa.

**Key points**

- Lennox–Gastaut syndrome (LGS) management requires regular assessments and multidisciplinary care.
- Polytherapy should be optimized, and new or emerging treatments explored.
- Family involvement is pivotal, with awareness and advocacy actively promoted.

## 1 INTRODUCTION

Lennox–Gastaut syndrome (LGS) is a severe developmental and epileptic encephalopathy (DEE) with a typical onset between the third and fifth years of age.^1^ The prevalence is ~1%–10% of all childhood epilepsies, and the incidence is ~0.6% of all new‐onset epilepsies, with a negligible male predominance.^2^ LGS was named after Dr. Lennox and Dr. Gastaut, who independently described the syndrome. The electroclinical features of the disorders include (i) multiple seizure types (of which tonic seizures are a hallmark feature); (ii) diffuse, slow spike‐and‐wave (SSW) <3 Hz, and generalized paroxysmal fast activity (GFPA) on electroencephalography (EEG) during sleep; and (iii) cognitive and often behavioral impairments, which may not be present at seizure onset.^3^, ^4^, ^5^ However, a universally agreed‐upon definition does not exist as the syndrome continues to evolve.^6^ As a matter of fact, SSW activity may no longer be present in adults where other abnormal activities could be detected; these could include, but are not limited to, diffuse bilateral background slowing, focal spike and wave discharges, multifocal epileptiform discharges, generalized polyspike and wave discharges, and generalized paroxysmal fast discharges.^7^ Or tonic seizures could potentially be absent at onset, replaced by epileptic spasms.^8^

LGS not only has neurobiological but also psychological and social consequences, which pose a significant burden on patients and their families/caregivers. The needs of patients and their families are often neglected in routine clinical practice. The workshop organized in Genoa, within the suggestive 14th‐century noble residence *Villa Quartara*, took place between November 28 and 29, 2024. The first day was divided into four main sessions, dedicated to diagnostic gaps, therapeutic advancements, and research efforts headed by the European Reference Network for Rare and Complex Epilepsies (ERN‐Epicare), with a focus on unified disease registries for Europe. The second day was chaired by family representatives and provided space for advocacy needs and non‐pharmacological treatment strategies.

## 2 KEY WORKSHOP SESSIONS

### 2.1 Advances in diagnostics

LGS is an electroclinical syndrome with high etiological variability. Causes may be identified in ~75% of cases, which are broadly divided into structural, genetic, metabolic, or combined (e.g., genetic *plus* structural) categories. The remaining cases have no identifiable underlying causes.^9^ However, the percentage of unsolved cases is decreasing due to the sophistication of investigations and the higher clinical ability to interpret results. Given the challenges associated with accurate LGS diagnosis, several key investigations are recommended. Identifying the underlying cause is crucial for selecting the appropriate treatment strategy.^1^

#### 2.1.1 Instrumental diagnostic tools

Given that LGS is an electroclinical syndrome, EEG is a cornerstone for a correct diagnosis. Overnight video‐EEG is mandatory for detecting GFAP (10–20 cycles/s) at the onset of the disease,^10^, ^11^ as well as in adults if a definitive diagnosis by an epilepsy specialist has not been previously achieved. Indeed, although EEG findings, such as slow background activity and diffuse SSW (DSSW) discharges during wakefulness, tend to normalize in adult life with a nearly normal standard EEG, GFAP tends to persist through adulthood^11^ and, coupled with tonic seizures during sleep, facilitates the diagnosis. However, as tonic seizures may sometimes manifest only with apnea, a thoughtful neurophysiologic investigation should include polysomnography with EEG, electromyography (EMG), and pneumography (PNG).^11^, ^12^

Modern 3‐T magnetic resonance imaging (MRI) scanners, equipped with sets of sequences for studying epilepsy, can identify subtle abnormalities, shedding light on previously inconclusive MRI scans.^8^ The Harmonized Neuroimaging of Epilepsy Structural Sequences (HARNESS‐MRI) protocol^13^ is applicable to both children and adults and includes isotropic, millimetric three‐dimensional (3D) T1 and fluid‐attenuated inversion recovery (FLAIR) images, as well as high‐resolution 2D submillimetric T2 images, all of which require no more than 30 min of acquisition. This protocol may increase lesion identification in 30%–65% of previously unremarkable MRI studies, and combined with image postprocessing, sensitivity may increase up to 70%.^13^

#### 2.1.2 Breakthroughs in genetic

Starting from 2013, with the publication in *Nature* of the extensive work by the Epi4K consortium within the Epilepsy Phenome/Genome Project, our understanding of the genetic basis of LGS has undergone a dramatic evolution. Multiple de novo single‐nucleotide variants (SNVs) have been associated with the disorder. These include mutations in genes such as *SCN1A*, *STXBP1*, *GABRB3*, *CDKL5*, *SCN8A*, *SCN2A*, *ALG13*, *DNM1*, and *HDAC4*.^14^ The diagnostic yield could be increased by including chromosomal microarray analysis to detect chromosomal abnormalities and copy number variants (CNVs).^15^ Understanding the molecular pathway in which each gene is involved, coupled with genotype–phenotype correlations, may direct the molecular diagnosis. For example, an abnormal brain MRI study, coupled with an electroclinical phenotype of LGS, may direct attention toward genes such as *DNM1*, *WDR45*, *DYNC1H1*, and *TSC1*.^14^, ^16^ Conversely, patients carrying mutations in *CHD2* (chromodomain helicase DNA‐binding protein 2) may show atypical EEG traits and conspicuous myoclonic seizures.^17^, ^18^

However, the genetic variation profiles between different epileptic phenotypes could overlap widely, as in many cases LGS could develop from infantile epileptic spasms syndrome (IESS).^19^ Consequently, the gene for LGS does not exist; instead, genes follow the mainstream of DEEs, where genetic heterogeneity (more genes for one phenotype) and allelic heterogeneity (more phenotypes for one gene) coexist. In clinical practice, understanding the genetic etiology is pivotal to guiding genetic counseling and the choice of precision therapies.

Neurometabolic disorders are a rare but worth investigating cause of LGS.^20^ Most of these diseases can already be identified through genetic testing. However, metabolic testing, including blood and urine tests, as well as, in selected cases, cerebrospinal fluid (CSF) analysis, can also be helpful in confirming or fulfilling the diagnostic criteria. For example, Leigh syndrome (or subacute necrotizing encephalomyelopathy) has been associated with ~14 mitochondrial‐related genes, with the *MT‐ND3* mutation being more consistently associated with an epilepsy phenotype.^21^ The consequences of impaired energy metabolism are reflected systemically, with specific signal alterations in the brainstem and basal ganglia on brain MRI, often associated with leukodystrophy and cerebral atrophy. Biochemical investigations include the assay of pyruvate dehydrogenase in leucocytes or cultured skin fibroblasts, as well as the assessment of oxidative phosphorylation in muscle or liver. Finally, lactate levels are invariably elevated in the CSF, and often also in the blood.^22^, ^23^

#### 2.1.3 Biomarkers and early detection methods

In recent years, significant strides have been made in identifying disease biomarkers, and, thanks to advanced data integration analyses, these biomarkers can now be utilized for early diagnostic purposes or to predict responses to treatments. EEG patterns are among the most studied and known disease predictors. The appearance of SSW activity, even in seizure‐free children, is a red flag for the development of LGS in at‐risk subjects,^24^ whereas deep learning analysis of scalp EEG may enhance the early detection and overall burden of GPFA.^25^ Laboratory biomarkers are also being investigated and are likely to become increasingly relevant; DNA methylation signatures (or “episignatures”), which are patterns shared among individuals with pathogenic variants in the same gene, have demonstrated to be useful for diagnostic purposes in genetically unresolved DEEs.^26^ Blood‐derived DNA analysis could be particularly relevant for ubiquitously expressed epilepsy genes, such as *CHD2*, whereas robustness decrease for those genes that are predominantly expressed in the central nervous system (CNS) such as *SCN1A*, for which a clear DNA methylation signature does not seem to exist.^27^

A new avenue of research is the microbiota–gut–brain axis and its associated biomarkers. Although the influence of the gut microbiota on neurodevelopment and predisposition to both systemic and CNS disorders has been known for ages,^28^ in the 20th century, with the advent of next‐generation sequencing and metabolomics for the identification of all detectable small molecules (also called metabolites) in a biological system,^29^ research has decidedly increased. Studies have attempted to identify bacterial fingerprints specific to certain epileptic syndromes or predictive of response to treatment. Nowadays we know that certain bacterial species, such as butyrate‐producing bacteria, could be predictive of treatment response in patients with epilepsy^30^, ^31^; similarly, butyrate, a short chain fatty acid, could be dosed in peripheral blood samples and could promptly reach the CNS, trespassing on the blood–brain barrier (BBB) through the specific monocarboxylate/sodium monocarboxylate transporter (MCT/SMCT), and protecting the brain and enhancing plasticity.^32^

Other CNS‐associated inflammatory biomarkers include the glial‐derived mediators, such as glial fibrillary acidic protein (GFAP), high mobility group box 1 (HMGB1), chitinase‐3‐like protein 1 (CHI3L1), soluble CD163 (sCD163), and triggering receptor expressed on myeloid cells 2 (TREM2). Patients with LGS can show higher serum levels of HMGB1, CHI3L1, sCD163, and TREM2 compared to other DEEs (i.e., West syndrome) and healthy controls. Moreover, most of these biomarkers showed the potential to serve as prognostic and predictive biomarkers: GFAP and TREM2 were associated with treatment response; HMGB1, CHI3L1, and sCD163 were higher in patients with severe epileptic encephalopathy; levels of CHI3L1 correlated linearly with the severity of motor/mental impairment and electrophysiological features, coming into prominence as a glial mediator and a potential biomarker associated with clinical severity.^33^ Further studies will validate these biomarkers and translate them into routine clinical practice to predict disease progression and response to treatment.

### 2.2 Treatment strategies

#### 2.2.1 Overview of established therapies and repurposed drugs

Current pharmacological treatments are purely symptomatic, with very few exceptions for cases secondary to genetic variants.^34^ Therefore, at present, the primary goal of treatment is to minimize the occurrence of disabling seizures, enhancing the quality of life (QoL), rather than aiming for the unlikely outcome of complete seizure freedom.^35^

The natural course of the disease fluctuates over time, making it essential to re‐evaluate the goals and treatment at least once/twice a year, depending on the clinical condition. Reducing generalized tonic–clonic seizures (GTCS) and drop attacks (DAs), due to the risks of sudden unexpected death in epilepsy (SUDEP) and injury, as well as preventing complications such as non‐convulsive status epilepticus (NCSE), are crucial.

Several randomized controlled trials (RCTs) have been conducted over the past 30 years to evaluate the efficacy (and safety) of different antiseizure medications (ASMs) for the treatment of LGS‐associated seizures. Given the refractory nature of the condition, these are add‐on ASMs trials rather than monotherapy ASM trials.^36^ Their results have led to the specific U.S. Food and Drug Administration (FDA) and European Medicines Agency (EMA) approval of felbamate (FLB), lamotrigine (LTG), topiramate (TPM), rufinamide (RUF), clobazam (CLB), and, most recently, cannabidiol (CBD) and fenfluramine (FFA) as add‐on treatment of LGS‐associated seizures.^34^, ^37^ However, other ASMs not specifically approved for LGS are also widely used in clinical practice, such as valproate (VPA).^38^ For example, the 263 patients recently enrolled in the FFA RCT, conducted from 2017 to 2019, had previously tried a median of 7 ASMs (ranging from 1 to 20) before entering the study.^39^

Currently, expert panel recommendations suggest categorizing the pharmacological treatment of LGS into three levels.^7^, ^35^

Although not specifically licensed for LGS, VPA is the most commonly used first‐choice drug. This is due to its broad spectrum of action, including in atypical absences and myoclonic seizures, its affordability, and its diverse formulations. In monotherapy and/or in combination with LTG, with which it has a synergistic effect,^40^ it is suggested as a first‐line treatment for LGS‐associated seizures. VPA has a well‐documented teratogenic potential; hence, guidance recommends avoiding it in women of childbearing potential. However, if other options have failed, individualized risk–benefit considerations can be made, and the treatment initiated under strict measures (i.e., the lowest effective dose, contraception, preconception counseling, etc.).^6^, ^7^, ^35^

Rufinamide represents a valid alternative in case of failure of the VPA–LTG combination, possibly replacing one of the two. When a new ASM in introduced, another should be gradually withdrawn. This approach ensures that polytherapy includes no more than two or three ASMs simultaneously.^35^

TPM and CLB are considered among the primary choices, although they are limited by lesser tolerability. TPM can negatively affect cognitive function, whereas benzodiazepines (BZDs) are suggested to be administered in cycles, typically during cluster seizures or NCSE, to minimize the risk of sedation and dependency.^35^ The first‐line tier includes CBD, also for its benefits on non‐seizure outcomes, whereas FFA has not yet been incorporated into treatment algorithms following its recent market approval.^7^, ^34^, ^38^

The Tier 2 category comprises levetiracetam (LEV), zonisamide (ZNS), and perampanel (PER), due to their broad spectrum of action and easy handling.^7^, ^34^, ^38^ The effectiveness of add‐on PER in LGS was investigated recently in a dedicated RCT, which, however, was conducted during the coronavirus disease 2019 (COVID‐19) pandemic and was prematurely terminated due to lack of recruitment.^38^

Other options include brivaracetam (BRV), lacosamide (LCM), and cenobamate (CNB), which may be useful for patients with recurring focal seizures and are part of the Tier 3 level.^7^, ^38^, ^41^

FLB is not available in Canada, Australia, or the United Kingdom, and it carries a high risk of aplastic anemia and hepatic toxicity. Therefore, it is commonly considered a Tier 3 drug. In addition, ethosuximide may play a limited role in the treatment of atypical absences, whereas small doses of phenobarbital may be used to control severe GTCS.^35^

The introduction of any ASM can potentially worsen epileptic seizures. In the population of patients with LGS, a worsening of seizures, particularly myoclonic, GTCS, and atypical absences, has been observed more frequently with carbamazepine, oxcarbazepine, eslicarbazepine, phenytoin, tiagabine, pregabalin, gabapentin, and vigabatrin. Therefore, these drugs should be used with extreme caution in very select cases.^7^, ^35^

Short steroid courses, along with BZDs, may be useful for treating exacerbations, although relapse is common.^35^

#### 2.2.2 Insights into surgery, neuromodulation, dietary therapies, and investigational drugs

Only a minority of patients are eligible for a potentially curative resective surgery, which, whenever possible, represents the first choice. The best candidates for a pre‐surgical workup are those who present with an asymmetrical EEG pattern congruent with a focal lesion. However, this option should not be overlooked, even if the EEG is diffusely abnormal.^42^, ^43^

Corpus callosotomy is technically a type of resective surgery, although with palliative purposes. It represents a viable strategy in the presence of frequent and disabling DA, due to its effectiveness on tonic and atonic seizures. In addition, with the introduction of laser interstitial thermal therapy, a reduction in procedural complications has been observed.^44^

Neuromodulation techniques also fall under the category of palliative surgery, albeit with a different approach. The experience with the vagus nerve stimulation (VNS), labeled from the age of 4 and widely available worldwide, has been well established for over two decades. Literature reports that approximately half of the patients “respond” to VNS, with an estimated 50% reduction in seizures.^45^ The greatest benefits appear to be for myoclonic seizures and absences, whereas the effect is moderate for focal and tonic–clonic seizures.^43^

Stimulation of the centromedian thalamic nucleus (CMT) with deep brain stimulation (DBS) has been shown to result in an estimated 50% reduction in seizures during the ESTEL trial.^46^ This technique is approved only for adults in both Europe and the United States, although its availability may vary by institution due to its technically more challenging nature from a neurosurgical perspective. Reported experience so far suggests a particular benefit for GTC, atonic, and absence seizures. Long‐term studies are ongoing.^43^

Finally, the responsive neurostimulation (RNS), which has the advantage of functioning as a “closed‐loop system,” is approved only in the United States for the treatment of drug‐resistant focal seizures in adults. It is under investigation in a trial involving patients 12 years of age or older with LGS, through bilateral stimulation of the CMT and the bilateral prefrontal cortex.^47^

Moving on from surgical interventions, dietary therapy is a well‐established non‐pharmacological treatment, largely used in pediatrics. The ketogenic diet (KD) showed seizure reduction rates comparable to many ASMs in several RCTs for LGS, estimated at ~30%–50%. The best candidates are individuals who require a gastrostomy, accompanied by highly motivated families who are well supported by a dedicated team. The potential efficacy is indeed highly dependent on the patient's strict adherence.^35^, ^48^

Currently we have no knowledge of biomarkers that can predict the response to non‐pharmacological treatment. Therefore, the choice must be individualized on a case‐by‐case basis. Relevant factors in the decision may include the availability of the technique, patient/caregiver preference, and the potential impact on the most prevalent and disabling seizure type. For the future, specific SNPs in genes relevant to the metabolism of a high‐fat, low‐carbohydrate diet may also guide the choice of patients to be treated properly.^49^

All of the above‐mentioned strategies are not mutually exclusive and can be combined and/or used sequentially.

Several investigational drugs are in clinical development for LGS. Soticlestat (TAK935), a first‐in‐class inhibitor of cholesterol 24‐hydroxylase, was recently investigated in the LGS population in a Phase 2 (ELEKTRA trial) and Phase 3 trial (SKYWAY trial), not demonstrating statistically significant efficacy compared to placebo in the respective endpoints (drop seizures and major motor drop).^50^, ^51^

Bexicaserin (LP352) is a highly selective serotonin 2C (5‐HT2C) receptor agonist that demonstrated a 51% improvement (compared to 17% for placebo) in motor seizures among 29 patients with LGS (including 5 on placebo) in the Phase 1b/2a PACIFIC Study (NCT05364021). Twenty of these patients continued in the open‐label extension for ~6 months.^52^, ^53^

The efficacy of carisbamate (RWJ 333369), a new oral neuromodulator, is being evaluated in pediatric and adult populations with LGS through a Phase 3 trial (DISCOVER study YKP509C003).^54^

#### 2.2.3 Risks of polytherapy

Beyond disease symptoms, every ASM itself has anticipated side events, either systemic (e.g., transaminase elevation) or on the CNS, which include potential negative effects on behavior, mood, cognition, sedation, and sleep.^55^ Among the second‐ and third‐generation ASMs used in the pharmacological treatment of LGS,^1^ current evidence suggests that TPM should be used, with awareness of possible language impairment and cognitive dulling/memory problems, whereas LTG and RUF could be associated with insomnia. Between broad‐spectrum ASMs, which can be useful for LGS treatment, LEV and PER, and to a lesser extent BRV,^56^ are associated with aggressiveness and irritability, whereas ZNS could be associated with negative effects in some aspects of cognition. Conversely, newer ASMs such as CBD and FFA may have some positive effects on patients' cognitive profiles, although evidence is still limited and under investigation. Finally, virtually all ASMs are associated with sedation, and the risk is increased by polypharmacological treatment, justifying the need to rationalize polytherapy and switching drugs instead of continuous add‐on.^1^, ^55^

Consequently, when choosing an ASMs, four main factors should be considered: (i) efficacy (i.e., is the drug appropriate for the types of seizures and epileptic syndrome of the patient?); (ii) tolerability (i.e., are the side effects are acceptable, or do they outweigh the benefit?); (iii) duration (i.e., what is the expected length of treatment?); (iv) individualization (i.e., is the drug appropriate for the patient considering its comorbidities and peculiar characteristics?).^57^ If the clinician could answer each of these questions, he could rationalize the treatment, avoiding wrong therapeutic choices and drug overload.

#### 2.2.4 How to design pharmacological clinical trials: choosing adequate outcomes and timepoints

Pharmacological trials for LGS are designed to assess primary endpoints such as “countable motor seizures” or “convulsive seizures” and DAs. The 2022 International League Against Epilepsy (ILAE) seizure semiology glossary refers to DAs as “astatic seizures,” when it is not possible to specify the myoclonic/atonic or tonic mechanism causing the sudden loss of maintaining the erect posture and, hence, the fall.^58^ At the same time, “convulsive seizure” or “motor seizure” can be interpreted by patients and caregivers as “anything that moves.” This heterogeneity may lead to difficulty in generalizing the study outcome.^59^ LGS presents with multiple seizure types, and the drug impact on each of them remains hard to assess in clinical trials.^59^ These insights are lost under the umbrella of “total seizure reduction.”

An ideal endpoint should be reliable, countable, and meaningful. Experts have proposed the adoption of “seizure‐free days” as a novel outcome that could more concretely correlate with an improvement in QoL.^59^ A “time to event” endpoint has also been suggested to adjust the baseline period of RCTs based on individual seizure burden in infants and young children.^59^

The further evolution of wearables for seizure detection (e.g., the Embrace watch) or devices for long‐term home EEG monitoring (e.g., subQ‐EEG) is expected to lead to precise quantification of seizures in the future, especially in the absence of witnesses and during nighttime hours.^60^, ^61^ Much discussion also surrounds the development and incorporation of “non‐seizure outcomes” or “patient‐centered outcomes” (such as sleep, behavior, and communication) into trials, given the profound developmental impact of LGS and the fact that many of the treatment strategies presented appear to provide benefits in these areas.^34^

### 2.3 Patient and caregiver perspectives

The burden of illness (BOI) in LGS encompasses outcomes including disease prevalence, clinical symptoms, patient and caregiver health‐related QoL (HR‐QoL), as well as health care resource utilization and costs.^59^ Investigating the BOI allows a comprehensive understanding of the challenges posed by the disease for patients/caregivers, the health care system, and society. Although seizures are invariably a huge determinant of the BOI, with seizure‐free days determining higher health care resource use, increased costs, and lower HR‐QoL.^62^ Patients with LGS generally have between 2.6 and 3.6 seizure types *per individual* and 50%–100% of them show daily seizures, with DAs having the greatest impact on the one hand on patients who may need to wear helmets to avoid physical injuries and caregivers' physical assistance, and on the other hand on parents/caregivers who needs to take days off work.^63^ Seizures tend to reduce over the years, with few patients reaching seizure freedom. Yet, patients and families come to face other challenges, and the so‐called “non‐seizure symptoms,” including cognitive and behavioral disorders and sleep disturbances, take over.^63^ Developmental delay/intellectual disability has long been considered one of the three symptoms of the LGS diagnostic triad. Although such criteria are nowadays less restrictive, a percentage between 80% and 100% of patients with LGS show moderate‐to‐profound delay in psychomotor development or intellectual disability.^1^, ^63^ Behavioral problems include autism spectrum disorder (ASD) and attention‐deficit/hyperactivity disorder (ADHD). Sleep disruption, including increased sleep‐onset latency and instability of sleep stages, as well as nighttime awakenings associated with seizure occurrence, can significantly impact both patients and their caregivers, reducing energy for daytime activities and affecting school and work performance.^64^

Although non‐seizure outcomes contribute significantly to overall perceived negative HR‐QoL from both a patient's and caregiver's perspective, there is a lack of comprehensive studies on their effect on patients, families, and society, and evidence is mainly based on systematic literature review.^65^ One major bias in this evaluation is the lack of unique assessment tools. In 2023, Bliss and colleagues made a collective effort in partnership with the Lennox–Gastaut Syndrome Foundation (LGSF) to gain first insight into patient and family experiences through a custom‐made survey tool on behavior, communication, and QoL. Caregivers express the relevance of assessing these areas and the perceived lack of comprehensive evaluation during routine follow‐up visits.^66^ Of interest, most caregivers were not even aware if they had completed specific questionnaires to evaluate these areas in the past. The highest reported instruments were the Adaptive Behavior Assessment System (ABAS), the Communication and Symbolic Behavior Scales (CSBS), and the Aberrant Behavior Checklist (ABC). The QoL was mostly evaluated through the Pediatric Quality of Life Inventory (PedsQL) or its epilepsy module and the Patient‐Reported Outcomes Measurement Information System (PROMIS).^66^ LoPresti et al.^67^ also conducted a quantitative online survey to evaluate the emotional well‐being, perceived HR‐QoL, and working productivity in carers of adult patients with DEE. The validated questionnaires included were the Hospital Anxiety and Depression Scale (HADS), the Pediatric Quality of Life Inventory Family Impact Module (PedsQL FIM), and the Work Productivity and Activity Impairment General Health (WPAI:GH). In adulthood, the lack of self‐sufficiency in everyday life activities (e.g., dressing and self‐care support) consumes the caregivers' time and impairs their emotional well‐being and work productivity. In clinical practice, some useful scales include the Child Behavior Checklist (CBCL) to assess emotions, behavior, and social aspects, and the Beck Depression Inventory (BDI‐II) for evaluating depression.^68^, ^69^ Sleep can also be evaluated using various scales, including the Pittsburgh Sleep Quality Index (PSQI), the Insomnia Severity Index (ISI), the Epworth Sleepiness Scale (ESS), and the Patient Health Questionnaire‐9 (PHQ‐9). Each scale investigates a specific “feature” of sleep, such as sleep quality, insomnia, daytime sleepiness, and correlation with depressive symptoms.^70^ Based on the scope, the right scale should be given to patients/caregivers to investigate the outcome of seizures and their QoL.

#### 2.3.1 Addressing the challenges of transition

Transition from pediatric to adult care is another major source of anxiety for both patients and their caregivers/siblings. “Transitioning” does not only mean the formal handover from the child to the adult specialist, but it also comprises the planned movement of the adolescent toward age‐appropriate care and self‐responsibility.^71^ Unfortunately, in most cases, this process is negatively perceived; this is due mainly to practical uncertainties and lack of a structured process of handover, and to the loss of the “holistic” management of childhood care. Moreover, when the adolescent has intellectual disability, the question becomes: “Is it the patient who is transitioning or their parents?” In this case, health care providers should still cooperate with the parents, and further questions arise regarding the correct timing for care handover in this “special” population and the less integrated adult service.^72^ Although more than 10 years have passed since the widespread increase in the scientific literature about the pitfalls and needs in the transition of care in epilepsy, the topic remains one of great uncertainty and debate in clinical practice.^73^ Some hospitals have developed and efficiently implemented some transition epilepsy programs. In many other centers, due to either geographical (i.e., physical distance between the pediatric and adult care service) or informatic (i.e., lack of shared informatic datasets between hospitals) issues, as well as the fear for some adult neurologists looking out to the intricate care of childhood‐onset epilepsies,^74^ the transition into adult care still needs a clear shared action plan.

The transition from pediatric to adult care is a complex process, particularly regarding the designation of a support administrator/guardianship, one of the most important figures for individuals with disabilities.

There is no unified European legislation about the support administrator (Table 1), but there are supranational principles and recommendations concerning the legal protection of people with disabilities or reduced capacity to act (e.g., the Convention on the Rights of Persons with Disabilities).

**TABLE 1:** Summary of guardianship world legislation.

Country	Legal term	Main law/reference	Year/reform	Support vs substitution	Official source/PDF reference
IT Italy	*Amministratore di Sostegno*	Law No. 6/2004 (modifies Civil Code, Arts. 404–413)	2004	Support	Low n. 6/2004
ES Spain	*Medidas de Apoyo*	Ley 8/2021 (Reform of Spanish Civil Code)	2021	Full Support (CRPD‐based)	BOE‐A‐2021‐9233 (PDF)
DE Germany	*Betreuung*	Bürgerliches Gesetzbuch (BGB), §§1896–1908	1992/2023 reform	Support	BGB §1896–1908
FR France	*Tutelle/Curatelle*	Code Civil, Articles 425–515	Ongoing reforms	Mixed	Légifrance – Code Civil
GB UK	*Deputy/LPA*	Mental Capacity Act 2005	2005	Support via consent or court	UK Gov – MCA 2005
US USA	*Guardianship/Conservatorship*	Varies by State (e.g., UGPPA, state civil codes)	Ongoing reforms	Substitution (trend to SDM)	National Guardianship Association
CA Canada	*Substitute Decision Maker (SDM)*	Varies by province (e.g., Ontario: Substitute Decisions Act, 1992)	1992–2000s	Mixed	Ontario SDA
AU Australia	*Guardian/Administrator/SDM*	Varies by state (e.g., Victoria: Guardianship and Administration Act 2019)	2019 (Victoria)	SDM in progress	Victorian Legislation
JP Japan	*Koken'nin/Hozen'nin*	Adult Guardianship Law (1999), revised 2016	1999/2016	Mixed	Japan Law Translation

In Italy, the support administrator or the “*Amministratore di Sostegno* (AdS)” is a legal figure established under Law no. 6 of January 9, 2004, incorporated into the Italian Civil Code (Articles 404–413), aimed at protecting adults who, due to illness, disability, or physical or psychological impairment, are temporarily or permanently unable to manage their personal or financial affairs. Articles 405 of the Italian Civil Code define the powers and duties of an AdS concerning two areas (alternatively or jointly): (i) care of the person (including health choices) and (ii) care of assets (for example, administration of salaries, pensions, etc.). These rules should be defined considering the severity of patient disabilities and with total respect for autonomy, preserving a person's legal capacity as much as possible.^75^

Despite the presence of regulations, the designation of an AdS may encounter several difficulties, both legal and practical, such as the lack of legal representation due to bureaucratic delays (i.e., submission of the application).

#### 2.3.2 Strategies for empowering families through education and advocacy

Patient‐centered care, including the accurate sharing of knowledge with individuals with epilepsy and their families, educating them on how to properly manage seizures and behavioral problems, informing them about the risk of SUDEP, and helping to develop self‐management skills, is pivotal in making individuals partners in epilepsy management.^76^

Implementing educational programs^77^, ^78^ and participation of family representatives in scientific meetings could help reduce the burden of anxiety associated with epilepsy diagnosis and caregiving of patients with epilepsy. Nowadays family associations have an increasingly important role in the scientific and medical community, either to collect funding for research or to inform/support caregivers on their child's needs and the unmet needs of adults. As a matter of fact, the LGSF has set up a 5‐year (2024–2028) strategic plan, which is based on four pillars to improve the lives of patients with LGS: (i) support, empower, and educate (SEE) LGS; (ii) awareness and community building events (ACB); (iii) accelerate research (Rch); and (iv) Build and Strengthen Organization (BSO). The first goal will be achieved through a structured Families First program, including ambassadors/navigators, as well as monthly and online support groups.^79^

### 2.4 Global and national initiatives

The ERNs are networks of European hospital centers with a high level of expertise in the treatment of rare and complex diseases, such as LGS. The ERNs were launched in 2017 and, currently, ERNs cover the main clusters of rare, complex, and low‐prevalence diseases. The ERN EpiCare specifically works on rare and complex epilepsies to improve and increase diagnoses of the causes of rare epilepsies; enhance early identification of patients with treatable rare causes; increase access to specialized care; further develop and design innovative clinical trials for new ASMs through the European Collaboration for Epilepsy Trials (ECET); deliver full access to, and use of, early pre‐surgical evaluation and epilepsy surgery; and foster research on innovative diagnostic tools and causal treatments.^80^

Each month, the network organizes patient case discussions divided by focus into genetics, neuropsychology, drug management, and pre‐surgical evaluation. The participation of EU experts allows a high‐level case discussion for educational purposes. Moving in this direction, the ERN EpiCARE has launched numerous interactive educational webinars, updates on clinical practice guidelines, and collaborations with other ERNs and EU‐funded initiatives, such as the European Joint Programme on Rare Diseases (EJP RD) and SOLVE‐RD.^80^ Finally, but more importantly, the work with patient advocates from European Patient Advocacy Groups (ePAGs) has led to the creation of information leaflets on rare epilepsies, which are synthetic but informative pieces of material deriving from the collaboration of patient advocacy groups and health care providers, and patient‐centered clinical trials.^80^, ^81^

Disease registries are computerized datasets containing patients' personal details, genetic, and clinical data; their relevance in rare diseases lies in supporting the development of medical interventions and research plans.^82^ Putting together cases of rare and ultra‐rare disorders may help to understand the real prevalence and incidence of a certain disease, tracking down the natural history of the disease in view of seeing the changes provided by revolutionizing therapies (i.e., gene therapies). The Italian National Institute of Health offers consultancy services to enhance support and research for rare diseases, including assistance with regulatory access, registry creation, and the development of secure databases for data sharing.

### 2.5 Research gaps and challenges

#### 2.5.1 Initiatives for caregiver support

Caregivers and siblings of individuals with LGS shoulder a considerable burden, often navigating complex therapeutic regimens, behavioral issues, comorbidities, and bureaucratic challenges. Addressing their needs requires a systemic, multidimensional approach.

One priority is improving regulatory and administrative support. Families frequently encounter barriers to accessing reimbursed medications for rare epilepsies due to fragmented procedures and limited guidance. Establishing dedicated case managers or care coordinators within epilepsy reference centers could help streamline access to orphan drugs, disability benefits, and specialized services. The integration of trained social workers and legal‐administrative advisors into care pathways would also alleviate the hidden workload of caregivers.

At the psychological level, caregivers experience elevated rates of stress, depression, anxiety, and social isolation. Standardized assessment tools, such as the PedsQL‐FIM and the WPAI scale, have documented this impact. However, psychological care remains patchy or absent. Evidence supports the implementation of structured programs, including psychoeducation, group counseling, resilience training, and digital mental health resources tailored to the caregivers of patients with neurological disorders.^83^ In addition, family empowerment through targeted training on seizure management, medication administration, emergency planning, and transitions into adulthood can improve outcomes and reduce the perceived burden.

## 3 OUTCOMES AND RECOMMENDATIONS

**FIGURE 1:**
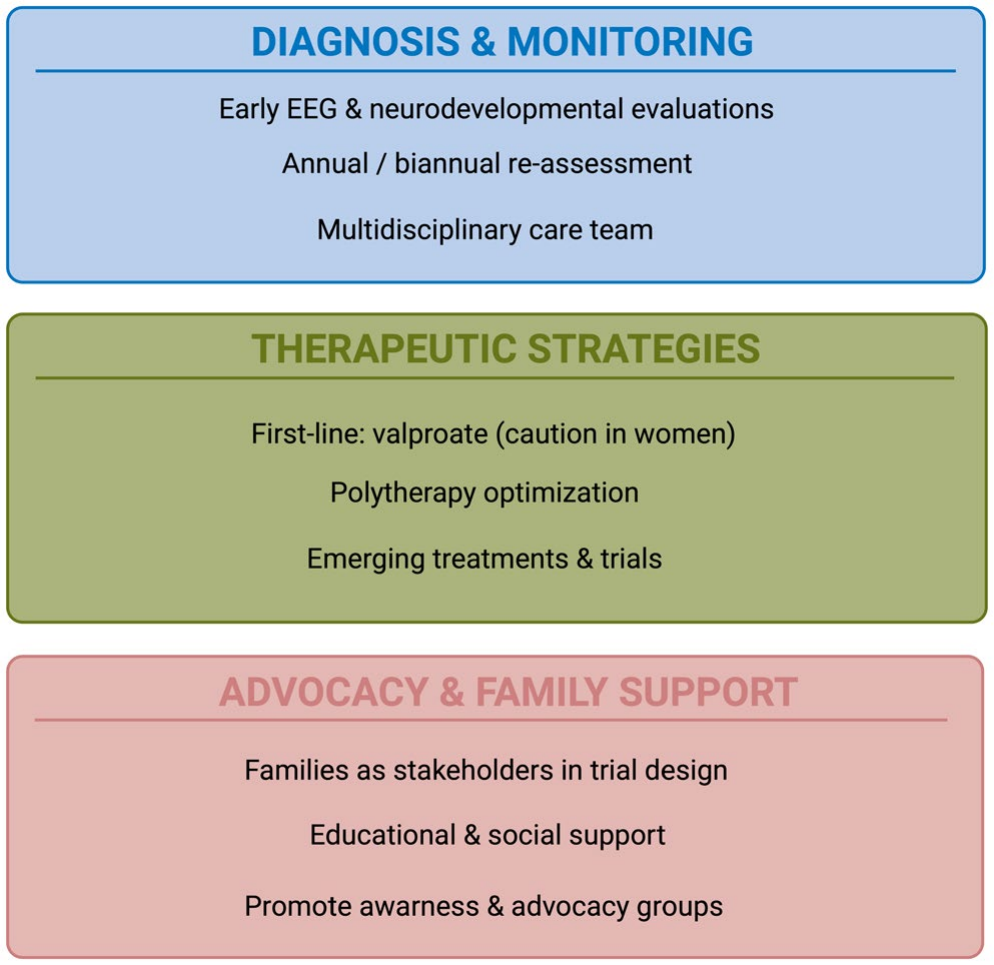
Care and research priorities in Lennox–Gastaut syndrome (LGS). EEG, electroencephalography. Created with Biorender.com.

The Genoa International Workshop became a space for listening, dialogue, and co‐construction, where clinicians, researchers, and families came together to share experiences and collectively envision a new direction for LGS care and research (see Figure 1). The presence of families and patient representatives was central. Their voices anchored the discussions in the realities of everyday life with LGS: drug‐resistant treatment, complex non‐seizure outcomes, uncertainty about the future, and the exhausting burden of navigating fragmented systems of care.

One of the most significant outcomes of the workshop was the recognition that families should not be treated as passive recipients of care or research subjects, but rather as active co‐designers. Their input is essential to identify research questions that matter and outcomes that are meaningful. Seizure reduction, though important, is often not the only—or the primary—priority. Improvements in sleep, behavior, communication, and daily functioning are valued equally by families, yet are frequently overlooked in traditional clinical trials. Participants discussed how the inclusion of family representatives in the design of research protocols, ethics review boards, and outcome assessments could dramatically improve both the relevance and success of future studies.

The workshop also reinforced the need to move beyond rigid syndromic definitions. Although the triad of multiple seizure types, characteristic EEG patterns, and intellectual disability remains a useful clinical construct, it does not capture the biological and phenotypic diversity within the LGS spectrum. Genetic testing, including exome sequencing and CNV analysis, is increasingly uncovering underlying etiologies, some of which may respond to targeted interventions. Moreover, there is growing interest in identifying biomarkers (i.e., genetic, epigenetic, neurophysiological, or microbiota‐derived) that could inform prognosis, guide treatment selection, or predict drug response. Establishing longitudinal registries and biobanks that integrate clinical, neuroimaging, and biological data was identified as a key priority to support biomarker discovery and validation.

The transition from pediatric to adult care was another recurring theme during the meeting, with many families describing it as a moment of rupture. Adult neurologists are often less familiar with the specific challenges of LGS, including its cognitive, behavioral, and systemic manifestations. Participants called for the implementation of structured transition plans to be implemented early, ideally during adolescence, with joint sessions between pediatric and adult teams, clear care objectives, and active family involvement. Without a deliberate strategy, the transition risks becoming a source of distress and discontinuity, undermining years of coordinated pediatric care.

Equally urgent is the need to support those who care for people with LGS. Caregivers reported high levels of psychological distress, anxiety, and fatigue, compounded by administrative complexity and lack of institutional support. There was a clear consensus that psychological support for caregivers should not be an optional extra but a formal part of the care pathway. Services such as psychoeducation, counseling, peer support groups, and respite care must be integrated into multidisciplinary teams. In addition, administrative support (e.g., assistance with disability claims) can significantly alleviate caregiver stress.

Several participants emphasized the importance of transcending national silos and adopting a pan‐European approach to LGS care and research. Networks like ERN EpiCARE provide a unique platform for sharing expertise, harmonizing protocols, and fostering large‐scale studies that no single country could undertake alone. However, patient organizations must be given a central role in these initiatives.

## 4 CONCLUSIONS

The main agreed priorities of the workshop were: the inclusion of family voices in every stage of research and care design; the expansion of early genetic testing and biomarker development; the implementation of structured transition programs; and the formal recognition of psychological and administrative support as essential components of LGS management.

What emerged in Genoa was not just a roadmap for LGS; it was a renewed vision of how rare and complex epilepsies should be addressed.

## AUTHOR CONTRIBUTIONS

A.R., collection of data and drafting; G.D'O. and A.D.L., drafting; A.A., S.A., I.B., P.B., V.B., I.Br., G.C., A.C., C.D.B., G.D.G., E.F., A.G.N., G.G., S.L., G.J.K., G.K., M.M.M., C.M., L.N., E.P., M.P., E.R., A.R., A.R., E.R., K.S., S.S.B., L.S., J.S., A.V., F.V., M.V., C.V.S., N.Z., and F.Z., workshop participation, revision and final approval of the manuscript; P.S., conception of the workshop, and revision and final approval of the manuscript. All authors agree to be accountable for all aspects of the work.

## FUNDING INFORMATION

No targeted fundings to be reported. IRCCS Istituto ‘G. Gaslini’ is a member of ERN‐Epicare.

## CONFLICT OF INTEREST STATEMENT

Neither of the authors has any conflict of interest to disclose.

## ETHICS STATEMENT

We confirm that we have read the Journal's position on issues involved in ethical publication and affirm that this report is consistent with those guidelines. All methods were performed in accordance with the ethical standards as laid down in the Declaration of Helsinki and its later amendments or comparable ethical standards.

## PATIENT CONSENT STATEMENT

The present study does not include human subjects.

## Supporting information


Figure S1.

## Data Availability

The data that support the findings of this study are available in the article.
