# Supplementary material for: Lennox–Gastaut syndrome unveiled: Advancing diagnosis, therapies, and advocacy‐insights from the Genoa International Workshop
Source: Epilepsia. 2025 Oct 29;67(2):542–56. doi: 10.1111/epi.18696 (PMC12927679; doi:10.1111/epi.18696)
Supplement: Supplementary file 1 — Figure S1. [file EPI-67-542-s001.docx]

**Supplementary**


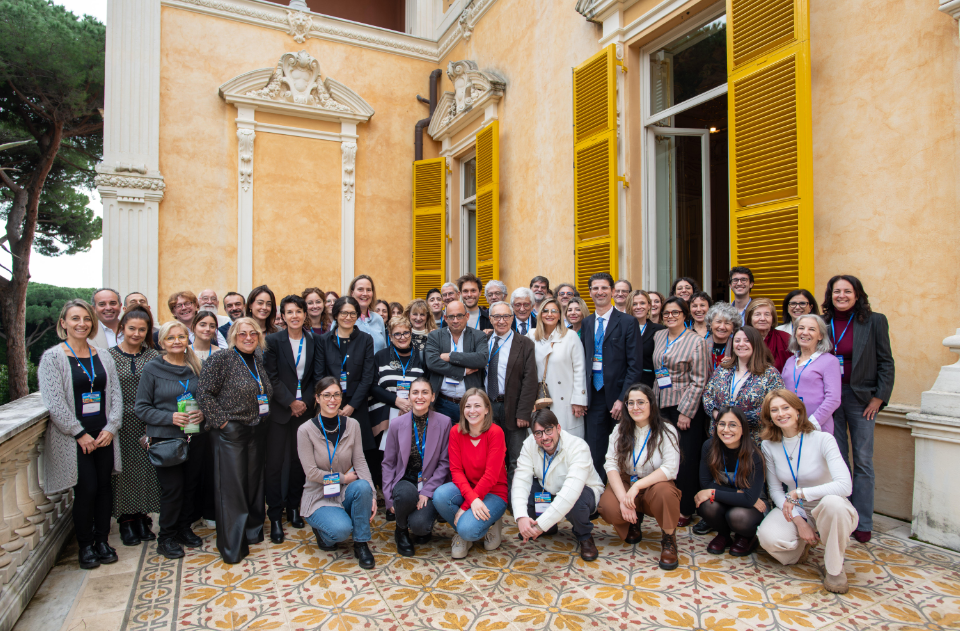


**Supplementary Figure 1.** Photograph of the participants at LGS Unveiled: Navigating Lennox-Gastaut Syndrome Together, held at Villa Quartara, Genoa, November 2024.
